# Integrated genomic surveillance enables tracing of person-to-person SARS-CoV-2 transmission chains during community transmission and reveals extensive onward transmission of travel-imported infections, Germany, June to July 2021

**DOI:** 10.2807/1560-7917.ES.2022.27.43.2101089

**Published:** 2022-10-27

**Authors:** Torsten Houwaart, Samir Belhaj, Emran Tawalbeh, Dirk Nagels, Yara Fröhlich, Patrick Finzer, Pilar Ciruela, Aurora Sabrià, Mercè Herrero, Cristina Andrés, Andrés Antón, Assia Benmoumene, Dounia Asskali, Hussein Haidar, Janina von Dahlen, Jessica Nicolai, Mygg Stiller, Jacqueline Blum, Christian Lange, Carla Adelmann, Britta Schroer, Ute Osmers, Christiane Grice, Phillipp P. Kirfel, Hassan Jomaa, Daniel Strelow, Lisanna Hülse, Moritz Pigulla, Pascal Kreuzer, Alona Tyshaieva, Jonas Weber, Tobias Wienemann, Malte Kohns Vasconcelos, Katrin Hoffmann, Nadine Lübke, Sandra Hauka, Marcel Andree, Claus Jürgen Scholz, Nathalie Jazmati, Klaus Göbels, Rainer Zotz, Klaus Pfeffer, Jörg Timm, Lutz Ehlkes, Andreas Walker, Alexander T. Dilthey, German COVID-19 OMICS Initiative (DeCOI), German COVID-19 OMICs Initiative (DeCOI), Janine Altmüller, Angel Angelov, Anna C. Aschenbrenner, Robert Bals, Alexander Bartholomäus, Anke Becker, Matthias Becker, Daniela Bezdan, Michael Bitzer, Conny Blumert, Ezio Bonifacio, Peer Bork, Bunk Boyke, Helmut Blum, Nicolas Casadei, Thomas Clavel, Maria Colome-Tatche, Markus Cornberg, Inti Alberto De La Rosa Velázquez, Andreas Diefenbach, Alexander Dilthey, Nicole Fischer, Konrad Förstner, Sören Franzenburg, Julia-Stefanie Frick, Gisela Gabernet, Julien Gagneur, Tina Ganzenmueller, Marie Gauder, Janina Geißert, Alexander Goesmann, Siri Göpel, Adam Grundhoff, Hajo Grundmann, Torsten Hain, Frank Hanses, Ute Hehr, André Heimbach, Marius Hoeper, Friedemann Horn, Daniel Hübschmann, Michael Hummel, Thomas Iftner, Angelika Iftner, Thomas Illig, Stefan Janssen, Jörn Kalinowski, René Kallies, Birte Kehr, Andreas Keller, Oliver T. Keppler, Sarah Kim-Hellmuth, Christoph Klein, Michael Knop, Oliver Kohlbacher, Karl Köhrer, Jan Korbel, Peter G. Kremsner, Denise Kühnert, Ingo Kurth, Markus Landthaler, Yang Li, Kerstin U. Ludwig, Oliwia Makarewicz, Manja Marz, Alice C. McHardy, Christian Mertes, Maximilian Münchhoff, Sven Nahnsen, Markus Nöthen, Francine Ntoumi, Peter Nürnberg, Stephan Ossowski, Jörg Overmann, Silke Peter, Klaus Pfeffer, Isabell Pink, Anna R. Poetsch, Ulrike Protzer, Alfred Pühler, Nikolaus Rajewsky, Markus Ralser, Kristin Reiche, Olaf Rieß, Stephan Ripke, Ulisses Nunes da Rocha, Philip Rosenstiel, Antoine-Emmanuel Saliba, Leif Erik Sander, Birgit Sawitzki, Simone Scheithauer, Philipp Schiffer, Jonathan Schmid-Burgk, Wulf Schneider, Eva-Christina Schulte, Joachim L. Schultze, Alexander Sczyrba, Mariam L. Sharaf, Yogesh Singh, Michael Sonnabend, Oliver Stegle, Jens Stoye, Fabian Theis, Thomas Ulas, Janne Vehreschild, Thirumalaisamy P. Velavan, Jörg Vogel, Sonja Volland, Max von Kleist, Andreas Walker, Jörn Walter, Dagmar Wieczorek, Sylke Winkler, John Ziebuhr

**Affiliations:** 1 Institute of Medical Microbiology and Hospital Hygiene, University Hospital Düsseldorf, Heinrich Heine University Düsseldorf, Düsseldorf, Germany; 2 Düsseldorf Health Authority (Gesundheitsamt Düsseldorf), Düsseldorf, Germany; 3 Institute of Virology, University Hospital Düsseldorf, Heinrich Heine University Düsseldorf, Düsseldorf, Germany; 4 Zotz | Klimas, Düsseldorf, Germany; 5 Sub-Directorate General of Surveillance and Response to Public Health Emergencies, Public Health Agency of Catalonia, Barcelona, Spain; 6 CIBER Epidemiologia y Salud Pública (CIBERESP), Instituto Salud Carlos III, Madrid, Spain; 7 Microbiology Unit, Vall d’Hebron University Hospital, Barcelona, Spain; 8 Medizinische Laboratorien Düsseldorf, Düsseldorf, Germany; 9 Solingen Health Authority (Gesundheitsamt Solingen), Solingen, Germany; 10 MVZ SYNLAB Leverkusen GmbH, Leverkusen, Germany; 11 Labor Dr. Wisplinghoff, Cologne, Germany; 12 Department of Hemostasis and Transfusion Medicine, Heinrich Heine University Medical Center, Düsseldorf, Germany; 13 Members of German COVID-19 OMICS Initiative (DeCOI) are listed under Collaborators.

**Keywords:** Genomic surveillance, contact tracing, deep backward contact tracing, travel-associated SARS-CoV-2 infections, SARS-CoV-2, Nanopore real-time sequencing, Next generation sequencing

## Abstract

**Background:**

Tracking person-to-person SARS-CoV-2 transmission in the population is important to understand the epidemiology of community transmission and may contribute to the containment of SARS-CoV-2. Neither contact tracing nor genomic surveillance alone, however, are typically sufficient to achieve this objective.

**Aim:**

We demonstrate the successful application of the integrated genomic surveillance (IGS) system of the German city of Düsseldorf for tracing SARS-CoV-2 transmission chains in the population as well as detecting and investigating travel-associated SARS-CoV-2 infection clusters.

**Methods:**

Genomic surveillance, phylogenetic analysis, and structured case interviews were integrated to elucidate two genetically defined clusters of SARS-CoV-2 isolates detected by IGS in Düsseldorf in July 2021.

**Results:**

Cluster 1 (n = 67 Düsseldorf cases) and Cluster 2 (n = 36) were detected in a surveillance dataset of 518 high-quality SARS-CoV-2 genomes from Düsseldorf (53% of total cases, sampled mid-June to July 2021). Cluster 1 could be traced back to a complex pattern of transmission in nightlife venues following a putative importation by a SARS-CoV-2-infected return traveller (IP) in late June; 28 SARS-CoV-2 cases could be epidemiologically directly linked to IP. Supported by viral genome data from Spain, Cluster 2 was shown to represent multiple independent introduction events of a viral strain circulating in Catalonia and other European countries, followed by diffuse community transmission in Düsseldorf.

**Conclusion:**

IGS enabled high-resolution tracing of SARS-CoV-2 transmission in an internationally connected city during community transmission and provided infection chain-level evidence of the downstream propagation of travel-imported SARS-CoV-2 cases.

## Introduction

Severe acute respiratory syndrome coronavirus 2 (SARS-CoV-2) has caused a worldwide pandemic with > 593 million cases and > 6.4 million associated deaths up to August 2022 [1]. SARS-CoV-2 vaccines have greatly contributed to reductions in coronavirus disease (COVID-19)-associated morbidity and mortality in many countries; however, non-pharmaceutical interventions (NPIs) to limit viral spread and reduce the healthcare burden of SARS-CoV-2 remain important in many contexts. Such contexts include instances of low vaccine availability or high rates of vaccine hesitancy in some countries, the potential for vaccine breakthrough infections and, more generally, the emergence of novel viral variants.

As the aim of NPIs is to interrupt or prevent pathogen transmission chains, a comprehensive understanding of these transmission chains in the population – who infected whom, and in which epidemiological context – could be greatly beneficial. Contact tracing regimes, which typically employ structured case interviews and which are operated by many countries, are an important data source on pathogen transmission in the population. Contact tracing is generally recognised as an important element of SARS-CoV-2 mitigation strategies [2-5]. However, the ability of classical contact tracing regimes to reliably track transmission chains in the population is limited and a substantial number of infections typically remain unexplained. For example, in the German city of Düsseldorf, an international economic and air travel hub of ca 600,000 inhabitants, ca 45% of SARS-CoV-2 infections remained unexplained in 2021 (Düsseldorf Health Department internal data), despite the operation of a well-staffed and comprehensive contact tracing effort. Similar numbers have been reported from other localities [6]. Genomic surveillance, another potential data source on the structure of population transmission chains, has also emerged as an important element of SARS-CoV-2 mitigation strategies [7,8]. However, due to the relatively low mutation rate of SARS-CoV-2 [9] and the fact that many genomic surveillance systems only sample a limited proportion of total cases, genomic surveillance by itself is typically not sufficient to enable reconstruction of transmission chains at the person-to-person level in the population outside of confined outbreak scenarios.

Integrated genomic surveillance (IGS) is an emerging approach that refers to the integrated analysis of genetic and complementary epidemiological data. As we and others have shown [10-13], IGS can contribute to identification of otherwise unrecognised SARS-CoV-2 transmission chains in the general population even under conditions of high-incidence community transmission and thus provide important complementary information for the design and implementation of NPIs.

Here we use the IGS system of Düsseldorf  (IGSD) to investigate person-to-person transmission chains in this city in late June and July 2021.

## Methods

### Integrated genomic surveillance in Düsseldorf

The IGSD has been described elsewhere [10]. Briefly, when fully operational, the system operates as follows. 

First, a large proportion of SARS-CoV-2 from local cases is rapidly sequenced. Viral genomes (Z* samples) are primarily generated by the Centre for Medical Microbiology, Hospital Hygiene, and Virology of Heinrich Heine University Düsseldorf as part of a dedicated local sequencing effort. Viral genome sequences of local cases generated under the national German SARS-CoV-2 surveillance programme by a collaborating large diagnostic laboratory (N* samples) are also integrated. In 2021, the achieved sequencing rate typically varied between 40 and 60% of new cases on a weekly basis; the ‘routine Düsseldorf surveillance dataset’ described below consists of sequence data obtained as described here (Z* and N* samples).

Second, putative infection clusters are identified with a search algorithm for groups of pairwise-identical samples (‘cliques’).

Third, the generated sequencing data and identified putative infection clusters are displayed in a visual form (‘dashboard’; available at https://covgen.hhu.de); this visualisation is continuously-updated. This system is used as the main information exchange mechanism with the Düsseldorf Health Authority.

Fourth, the identified putative infection clusters are investigated at the Düsseldorf Health Authority. The investigation combines (i) routine data collected as part of Düsseldorf Health Authority’s contact tracing activities, including on symptom onset, travel history and contact persons and (ii) information obtained from structured case interviews (‘deep backward contact tracing’; see below) to elucidate potential case connections not captured by standard contact tracing.

Of note, the IGSD does not comprise the routine collection of clinical metadata, and case severity is not used as a sample selection criterion.

To investigate the applicability of the developed system beyond Düsseldorf, a trial run of the IGS system was carried out in the nearby smaller city of Solingen in July and August 2021; the Solingen data were processed and analysed separately from the Düsseldorf data and only integrated with the Düsseldorf data during the phylogenetic cluster refinement analysis (see below).

### Inter-sample distance metric

Inter-sample genetic distances were calculated as defined previously [10], with one modification (point (iv) below). Briefly, a multiple sequence alignment (MSA) of all sequences was built with MAFFT [14], using GISAID [15] instructions. The distance d(x, y)  between two samples *x* and *y* was defined as the number of differences between the MSA entries of x and y , (i) ignoring leading or trailing gap characters, (ii) counting matches and mismatches according to International Union of Pure and Applied Chemistry (IUPAC) ambiguity codes, (iii) counting subsequent non-matching gaps columns as a difference of 1, (iv) ignoring deletions aligned to ‘N’ regions in the other genome, and (v) ignoring any mismatches in the MSA regions between the beginning of the MSA and the 20th ACGT character of either sequence and the end of the MSA and the 20 last ACGT characters of either sequence.

### Structured case interviews

A specialised team of interviewers within Düsseldorf Health Authority conducted structured case interviews. These covered (i) occupation and place of work; (ii) utilisation of public transport; (iii) social, household and family contacts; (iv) utilisation of medical services; (v) supermarket and retailer visits; (vi) gastronomy and nightlife; (vii) travel history. Before a case was classified as unavailable, a minimum of three contact attempts were carried out using available landline or mobile phone numbers; participation in the structured case interviews was voluntary.

### Phylogenetic cluster refinement analysis

**Figure 1:**
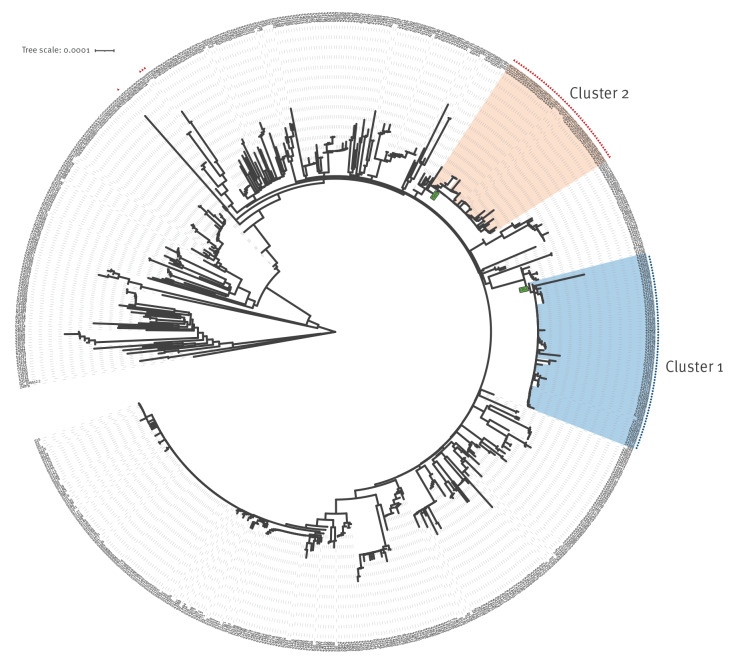
Phylogenetic analysis of the extended dataset of sequences of SARS-CoV-2 registered cases, Düsseldorf and Solingen, Germany, 15 June–01 August 2021 (n = 699 sequences)^a^

To refine the definition of two large groups of genetically-related-SARS-CoV-2-infected cases, which are referred to as Cluster 1 and Cluster 2, phylogenetic analysis of an ‘extended’ dataset (see Results section) was carried out using the neighbour-joining method with the Tamura–Nei genetic distance model as implemented in Geneious version 10.2.6 (Figure 1). The samples previously flagged by the IGSD routine cluster analysis algorithms were located in the phylogenetic tree. Once the presence of two large clusters of genetically-related isolates was confirmed, an analysis of the mutational patterns observed downstream of the putative cluster-associated root nodes was carried out. FASTA files and the phylogenetic tree are publicly available (see Data availability).

SARS-CoV-2: severe acute respiratory coronavirus 2.

^a^ The phylogenetic tree, generated with Geneious (see Methods) and visualised with iTol [19], shows 699 sequences. Nine sequences were found to exhibit low-quality alignments in the multiple sequence alignment of the 708 input sequences by manual inspection and were removed from the alignment before construction of the tree.

Cluster 1 and Cluster 2 are highlighted (areas of the tree shaded in blue and red, respectively). The nodes serving as the two clusters’ root nodes, I361 and I584, are displayed as little boxes with green background. The presence of T14064C and C18744T, mutations used in the process of defining Cluster 1 and Cluster 2, is indicated by blue circles and red triangles, respectively.

### Cluster 1 strain-of-origin analysis background dataset

To investigate potential origins of the viral strain of Cluster 1, a background dataset was assembled by combining (i) a random sample of non-Cluster 1 Düsseldorf sequences (n = 30); (ii) the set of all SARS-CoV-2 sequences from the Balearic Islands sampled between 15 June and 01 July 2021 available on GISAID (n = 173); (iii) the sequence of EPI_ISL_2710175, a viral genome from the Balearic Islands sampled on 14 June 2021 that was identified using the GISAID Audacity Instant Search [15]; (iv) 61 GISAID sequences related to Cluster 1. This GISAID set was assembled by carrying out a tree neighbourhood search in the GISAID ‘Global Phylogeny’ tree from August 2021 (GISAID-hCoV-19-phylogeny-2021–08–16; representing 624,052 sequences). Specifically, two Cluster 1 sequences (N1501, N1506) with genetic distance 0 to an individual SARS-CoV-2-infected traveller returning to Düsseldorf from the island of Mallorca (IP), who was retrospectively identified as a likely Cluster 1 index case in the city, were located in the tree. The identities of all leaves with a tree distance (defined as the cumulative length of the edges along the shortest path between two nodes) of ≤ 3/29,903 to either of the two Cluster 1 sequences and sampling date ≤ 15 July 2021 were extracted. 

The corresponding viral genome sequences were obtained from the GISAID MSA, and details and acknowledgements are provided in Supplementary Table 1.

## Results

### Detection and refinement of two large clusters in July 2021

Between 15 June and 01 August 2021, 541 SARS-CoV-2 surveillance genome sequences from Düsseldorf were registered within the IGSD, of which 518 were high-quality sequences (< 5,000 undefined nt; Supplementary Table 2); this set is referred to as the ‘routine Düsseldorf surveillance dataset’. Over the same period, 976 new SARS-CoV-2 cases were registered in Düsseldorf, i.e. a high-quality viral genome sequence was available for ca 50% of cases.

In mid-July 2021, the emergence of multiple overlapping putative infection clusters (‘cliques’ in the pairwise isolate distance matrix, i.e. groups of multiple pairwise-identical viral isolates) was detected by the system’s routine cluster analysis algorithms in the routine Düsseldorf surveillance dataset and indicated the presence of two novel large groups of closely related viral isolates (Delta variant; Phylogenetic Assignment of Named Global Outbreak (Pango) lineage designation: B.1.617.2 [16]).

Phylogenetic analysis (see Methods section; Figure 1) was carried out based on an expanded dataset, referred to as the ‘extended dataset’, with 708 viral genome sequences sampled between 15 June and 01 August 2021 (Supplementary Table 2) that comprised the original Düsseldorf routine surveillance dataset (n = 518); lower-quality Düsseldorf surveillance sequences (n = 23); Düsseldorf University Hospital patient sequences from the same period (n = 27); and available sequences from the nearby city of Solingen, where a trial run of the IGS system took place in July and August 2021 (n = 140). In this analysis, quality thresholds were applied after the construction of the phylogenetic tree; the rationale for including sequences from Solingen was to investigate potential transmission beyond Düsseldorf.

The phylogenetic analysis identified the mutation T14064C (blue circles in Figure 1) as associated with Cluster 1, and C18744T (red triangles) as associated with Cluster 2. The internal nodes I361 and I584 of the constructed phylogenetic tree (see Figure 1 and ‘Data availability‘) were chosen as the root nodes for Cluster 1 and Cluster 2 respectively. The isolate clusters Cluster 1 and Cluster 2 were provisionally defined as the sets of leaf-level descendants of these nodes, including Z4116, a sample carrying an isolated undefined genotype (‘N') at position 14064 with distance 0 to other Cluster 1 samples (e.g. IP).

Subsequent to the creation of the phylogenetic tree (Figure 1) with the extended dataset, phylogenetic outliers, repeat samples from the same individual, sequences with > 5,000 undefined nt, and non-surveillance Düsseldorf University Hospital sequences sampled after 09 July were removed (see Supplementary Table 3 for a full list of included and removed samples). For Cluster 1 (n = 71 leaf-level descendants of node I361 in the tree), this meant removing 11 sequences (two outlying – including a sequence from a case named KP1_5; four redundant; two with > 5,000 undefined nt – including one from a case named KP5_2_1; and three non-surveillance). For Cluster 2 (n = 49 leaf-level descendants of node I548 in the tree), this meant taking away seven sequences (one with > 5,000 undefined nt; four redundant; and two non-surveillance).

The phylogenetics-based definition of Cluster 1 comprised 60 viral sequences with an average pairwise genetic distance of 0.91 (59 from Düsseldorf, one from Solingen), sampled from 05 July onwards (Supplementary Table 4); of Cluster 2, 42 viral sequences with an average pairwise genetic distance of 1.89 (36 from Düsseldorf, six from Solingen), sampled from 30 June onwards (Supplementary Table 5).

The FASTA file used for the phylogenetic analysis comprising all analysed sequences as well as the constructed tree in Newick format are publicly available (see ‘Data availability‘). To further investigate Cluster 1 and Cluster 2, integration of routine contact-tracing data and structured case interviews were carried out (see Methods’ section).

### Cluster 1 was associated with nightlife spreading events following a putative travel-associated importation

**Figure 2:**
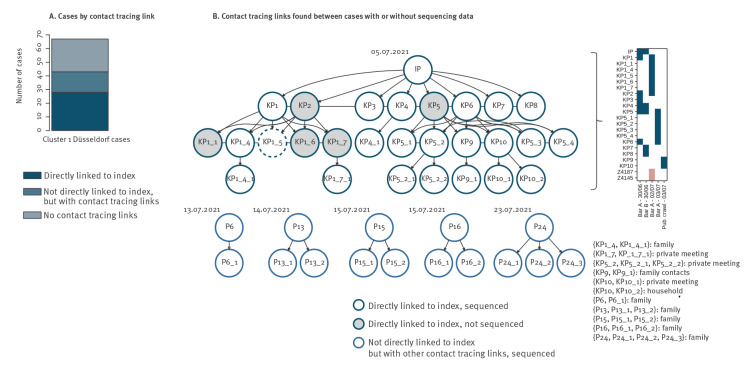
Cluster 1 contact tracing results, Düsseldorf and Solingen, Germany, 15 June–01 August 2021 (n = 68 cases)

The emergence of Cluster 1 in Düsseldorf could be traced back to multiple nightlife spreading events following putative importation of the Cluster 1-associated strain by IP, an individual SARS-CoV-2-infected traveller returning to Düsseldorf from the island of Mallorca on 28 June (the sequence of IP was not included to compile the phylogenetic tree in Figure 1 as this case was identified during the epidemiological investigation). The identified epidemiological links between Cluster 1 cases are visualised in Figure 2. Transmission of the imported viral strain (Delta variant) in Düsseldorf was likely initiated during encounters between IP and eight first-order contacts (KP1–KP8) in two bars (‘Bar A’, ‘Bar B’) in the Old Town District of Düsseldorf, a popular area for nightlife activities with narrow streets and more than 200 bars, on 30 June. Additional transmissions took place in a complex pattern of additional visits of the first-order contacts to Bar A on 02 July (KP2 and KP1 were present in the bar in the same time as KP1_1–KP1_7) and 03 July (Figure 2B Inset) during a likely encounter between the first-order contacts and KP9 and KP10, who were on a pub crawl in the area around Bar A on 03 July (where KP5 and KP6 were present, as well as KP5_1–KP5_4); and from the second-order contacts into the local population via private meetings, family and household contacts (Figure 2B). Contact tracing and structured interviews also uncovered links between an additional 15 cases without direct links to IP (Figure 2B); these likely represented ongoing community transmission of the introduced viral strain or secondary introduction events (see below). Apart from IP, the other cases had no recorded travel histories.

IP: infected return traveller, which is considered as the putative index case.

**A.** Bar plot showing (i) the number cases who had a direct contact-tracing link to IP (i.e. first-, second- and third-order contacts), (ii) the number of cases with no contact-tracing link to IP but with an epidemiological link to other Cluster-1 cases, who were also not related to IP and (iii) the number of cases with no contact-tracing links to any Cluster 1 cases.

**B.** Visualisation of the reconstructed epidemiological structure of Cluster 1. Each node in the transmission chain graph represents one case. Nodes shaded in grey represent epidemiologically linked, but non-sequenced cases (see text). Unshaded nodes represent epidemiologically linked and sequenced cases. Of note, the sequence of IP was not used for Figure 1, as this case and respective sequence data were found later in the investigation. While KP1_5 and KP5_2_1 cases had sequence data, these were initially not included in the phylogenetics-based definition of Cluster 1: KP1_5 exhibited an increased genetic distance to the other samples in the cluster (and is therefore shown with a dashed border; see Supplementary Note for a discussion) and KP5_2_1 had > 5,000 undefined nt. Upon contact tracing findings, however, these two cases were included as part of Cluster 1. Test dates of assumed index cases, who were epidemiologically unconnected to IP, but for whom contact-tracing suggested that they further transmitted the Cluster-1 strain to other IP-unconnected cases, are respectively shown above the respective nodes of these assumed index cases. The inset to the right of the transmission-chain graph shows the complex patterns of visits of IP and IP’s first- and second-order contacts to two bars in the Old Town District of Düsseldorf. The two cases shown with red boxes also visited Bar A, but their sequenced viral isolates group with Cluster 2 in phylogenetic analysis. Cases KP9 and KP10 participated in a pub crawl in the Düsseldorf Old Town area around Bar A. The text inset details the precise nature of the identified case relationships. Transmission chain graphs were plotted with Graphviz [20].

Of note, looking into a potential link between IP and Cluster 1 begun after it emerged during routine contact tracing that IP had frequented Düsseldorf Old Town nightlife venues on 30 June; when this link started to be explored, the investigation of Cluster 1 was already under way and had identified the Old Town and 30 June as focal points for Cluster 1-related viral transmission. The positive PCR test of IP was carried out in a laboratory not located in Düsseldorf and therefore not covered by the IGSD; the viral genome of IP (available on GISAID under EPI_ISL_3044996), however, was sequenced under Germany’s SARS-CoV-2 national genomic surveillance programme and could be requested by Düsseldorf Health Authority after identification of IP. Analysis of the viral genome sequence of IP confirmed that it was highly related to Cluster 1, carrying the T14064C mutation and exhibiting a genetic distance of 0 to 34 of the 60 Cluster 1 sequences phylogenetically defined (Supplementary Table 4).

The assignment of IP as the likely Cluster 1 index case was based on the reconstructed pattern of likely infection events as well as on the dates of symptom onset (Supplementary Table 4) of IP (01 July) and KP1–8 (04–07 July for all cases but KP1, who reported symptom onset on 02 July). IP’s symptom onset on 01 July rendered an infection on 30 June unlikely and favoured Mallorca or the return flight to Düsseldorf as infection contexts. In addition, apart from IP, only KP4 and KP5 were present in both Bar A and Bar B (Bar B was where KP7 and KP8 were likely infected) on 30 June, and KP4 and KP5 reported symptom onset on 04 July, consistent with an infection transmitted by IP on 30 June.

To further investigate potential origins of the viral strain of Cluster 1, we analysed the sequences of Cluster 1 against a background dataset of other contemporaneous sequences from Düsseldorf, the Balearic Islands, and GISAID samples related to Cluster 1 (see Methods section). Consistent with an assumed infection of IP on Mallorca, phylogenetic analysis (Supplementary Figure 1) showed that the sequences of Cluster 1 and a small number of isolates from the Balearic Islands and GISAID formed a distinct cluster. Furthermore, an analysis of genetic distances (Supplementary Table 6) showed that the Cluster 1-related sequences from the Balearic Islands were as closely related (genetic distance 1) to the sequence of IP as any of the GISAID sequences up to a sampling date of 07 July, approximately 1 week after the initiation of Cluster 1 transmission in Düsseldorf. IP-identical viral isolates started appearing in the GISAID dataset with sampling dates from 07 July onwards; the ‘originating laboratory’ record of the earliest three such isolates, however, indicated a likely sampling location in the area around Düsseldorf and thus a likely connection to Cluster 1. The first IP-identical isolates in the GISAID dataset from another German state were collected from 13 July onwards; these, as well as earlier Cluster 1-related sequences from June and July with genetic distance 1, may reflect wider circulation of Cluster 1-related strains in Europe and highlight the possibility of independent introduction events, as well as, in particular for the GISAID samples collected from mid-July onwards, potential export of the Cluster 1 viral strain from the Düsseldorf area.

**Figure 3:**
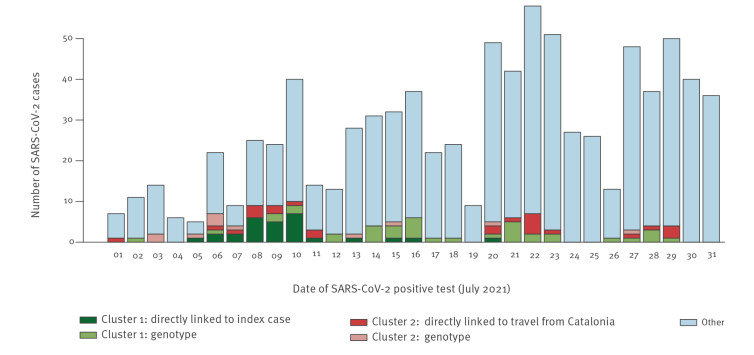
Distribution of SARS-CoV-2 cases according to time, their cluster, and epidemiological or genomic basis for inclusion in a cluster, Düsseldorf, Germany, 01 July–31 July 2021 (n = 850 cases)

IP: infected return traveller, which is considered as the index case; SARS-CoV-2: severe acute respiratory coronavirus syndrome coronavirus 2.

Including IP; two individuals (KP2, KP5) who were in the company of KP1, KP3, KP4, and KP6 when they were likely infected by IP; three individuals (KP1_1, KP1_6, KP1_7) who were with KP1_4 and KP1_5 when they were likely infected by KP1 or KP2; KP5_2_1 (the viral genome of whom had more than 5,000 undefined nt and who was therefore removed from the initial results of the phylogenetic analysis); and KP1_5 (the viral genome of whom exhibited an increased genetic distance to the other samples in the cluster; see Supplementary Note), 28 SARS-CoV-2 cases in Düsseldorf could be directly linked to IP (defined as the identification of an uninterrupted, contact tracing-supported putative transmission chain between the linked cases and IP; Figure 2A), with a median serial interval of 3 days. With these cases included, Cluster 1 comprised 67 Düsseldorf cases, or 8% of new SARS-CoV-2 cases registered in Düsseldorf in July (Figure 3), and one Solingen case. Of note, two cases belonging to Cluster 2, Z4187 and Z4145, also visited Bar A on 02 July (without recorded direct contacts to other Cluster 1 cases); despite this potential epidemiological link, the genetic data clearly showed that these belonged to a different infection cluster. For 24 Düsseldorf cases and S88, the only Solingen sample in Cluster 1, no links to other Cluster 1 samples or other putative infection sources were identified.

The figure shows daily new registered SARS-CoV-2 cases in Düsseldorf in July 2021, and how many of these were associated with Cluster 1 or Cluster 2. For Cluster 1, ‘directly linked to index case’ refers to uninterrupted, contact tracing-supported putative transmission chains between the linked cases and IP; ‘genotype’ refers to respective case samples that were identified as belonging to Cluster 1 in the phylogenetic tree analysis (see text). For Cluster 2, ‘directly linked to travel from Catalonia’ refers to cases who either recently returned from Catalonia and to cases who were directly linked to Catalonia returnees in a manner supported by contact tracing; ‘genotype’ refers to respective case samples that were identified as belonging to Cluster 2 in the phylogenetic tree analysis (see text).

Investigations by the Düsseldorf Health Authority and the discovery of a video posted to social media channels showed limited adherence to mandatory SARS-CoV-2 infection prevention measures in Bars A and B in force at the time, including dancing and non-compliance with indoor masking rules. In addition, the investigations demonstrated insufficient tracking of customer contact details, also in violation of mandatory German pandemic regulations in place at the time.

### Cluster 2 represents multiple independent importation events linked to return travel

**Figure 4:**
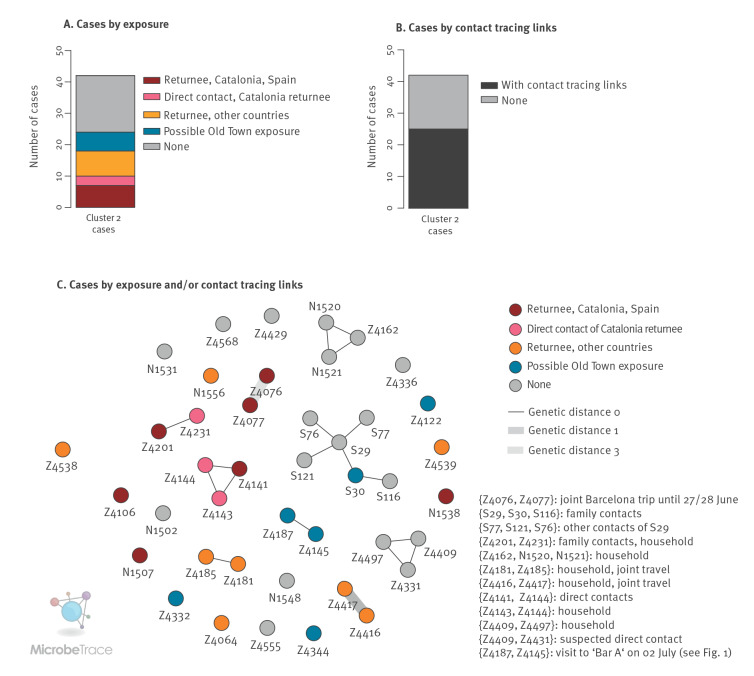
Cluster 2 genetic structure and contact tracing results, Düsseldorf and Solingen, Germany, 15 June–01 August 2021 (n = 42 cases)

For Cluster 2, detected from 30 June onwards and comprising 42 cases, no clear index case could be identified. While the integration of contact tracing data and structured case interviews enabled delineating relationships for 25 cases (e.g. household contacts), the overall size of the identified transmission chains was limited compared with Cluster 1 (Figure 4A). Examination of the travel history of the cases, however, showed that almost a quarter of the 42 Cluster 2 cases could be linked to return travel from Catalonia (seven returnees from Catalonia and three associated downstream infections in Düsseldorf); an additional five cases had been travelling to France before testing positive for SARS-CoV-2 (Figure 4B, Supplementary Table 5). Analysis of symptom onset (Supplementary Table 5) in relation to return travel dates suggested that eight of 15 return travellers in Cluster 2 were likely infected during their stay abroad because their symptoms had started either when abroad or within the first 24 hours after return. For some of these cases, exposure in Düsseldorf could be ruled out with certainty (e.g. Z4106, with symptom onset on the same day as the return flight). The viral sequences of Z4077 and Z4076, which belonged to some of the earliest Cluster 2 cases in Düsseldorf, exhibited a genetic distance of 3. The cases corresponding to these two sequences were likely infected during a joint trip to Barcelona (symptom onset 1 and 3 days after return to Düsseldorf, respectively), and likely represented two independent infection events in Barcelona. Cluster 2 thus likely reflected multiple independent introduction events of a viral strain also circulating in Catalonia, Spain and other European countries, followed by diffuse community transmission in Düsseldorf. Consistent with this, isolates closely related to Z4076 (EPI_ISL_2982413; genetic distance 1; sampling date 30 June) and Z4077 (EPI_ISL_3306421, EPI_ISL_3009886, EPI_ISL_3009934; genetic distance 0; sampling dates from 06 July onwards) were identified by genomic surveillance in Barcelona, in a collaborative effort to investigate this cluster. Of note, an additional direct contact of Z4077 and Z4076 in Barcelona also tested positive for SARS-CoV-2 after return to Düsseldorf and reported many contacts at work and in social gatherings in Düsseldorf and other cities, possibly contributing to community transmission; the viral sequence of this case could be obtained through a commercial diagnostic laboratory in Cologne and was found to also cluster with Cluster 2 (data not shown). Consistent with diffuse community transmission in and around Düsseldorf, for five additional cases from Düsseldorf, and one case from Solingen at the beginning of a six-person transmission chain, case interviews suggested a possible exposure to the virus in the Old Town district of Düsseldorf (Figure 4). Furthermore, consistent with circulation of the Cluster 2 viral strain in other European regions, sequence-identical samples from multiple European countries (e.g. Belgium France, Germany) were identified via a GISAID Audacity Instant search for the sequence of Z4077, with sample collection dates of the identified sequences beginning on 24 June, i.e. approximately 1 week before sample collection of the first Cluster 2 cases in Düsseldorf (30 June). Over the course of July, Cluster 2 accounted for 4% of total SARS-CoV-2 infections in Düsseldorf.

**A.** Bar plot showing the number of cases according to their status with regard to travel, contacts with returning travellers and exposure to the Old Town of Düsseldorf in Cluster 2.

**B.** Bar plot showing the number of cases in Cluster 2 with contact-tracing links found to other cases in Cluster 2 and the number of cases with no links identified by contact tracing.

**C.** Visualisation of genetic distances and contact tracing results for Cluster 2, created with MicrobeTrace [21]. Each node represents one case; edges between nodes indicate links identified by contact tracing; edge colours indicate genetic distances between the samples linked by contact tracing. The text inset details the precise nature of the identified case relationships.

## Discussion

Increased understanding of SARS-CoV-2 transmission chains in the population is important to support improved containment strategies. Here, we used IGS, an emerging approach integrating genetic and classical epidemiological data, to investigate two large clusters of genetically-related SARS-CoV-2 isolates occurring in Düsseldorf. Taken together, isolates from these two clusters accounted for more than 10% of SARS-CoV-2 cases in the city during the considered period (Figure 3). We show that IGS allowed to trace complex SARS-CoV-2 transmission chains in both clusters, each involving cases who had journeyed abroad. 

While both identified clusters were related to travel-imported SARS-CoV-2 infections, they exhibited different patterns with respect to transmission and potential public health implications. A large proportion of detected cases in Cluster 1 could be linked to a putative index case, who was a returning traveller with an initially undetected SARS-CoV-2 infection; non-adherence to infection prevention rules at nightlife venues contributed to an environment conducive to subsequent spread of infections. Cluster 1-associated case load could likely have been reduced at multiple points: by detecting the infection of IP upon return to Germany e.g. through a PCR test at the border; by mandatory quarantine regardless of infection status for return travellers; or by measures to reduce infection risks in nightlife settings, such as mandatory PCR or rapid antigen testing of patrons or strict adherence to physical distancing and indoor masking rules. What is more, compliance with mandatory customer contact data collection rules in place would have benefited post-hoc cluster investigation efforts, and it may also have contributed to further infection containment efforts. Of note, Spain, including the Balearic Islands, was only declared a region of high COVID-19 risk by the German health authorities on 27 July [17], i.e. almost 4 weeks after the first transmission in the reported clusters occurred. Cluster 2, by contrast, was driven by multiple independent introductions of a viral strain also circulating in Catalonia and other parts of Europe, and a larger number (n = 6) of Cluster 2 cases were found in another city, Solingen, which is part of the wider metropolitan area around Düsseldorf. Strict testing requirements for return travellers would likely also have contributed to a significant reduction of Cluster 2 cases.

IGS was a necessary approach for the investigation of the two clusters; in many instances, links between cases were only uncovered by the structured case interviews carried out after genetic links had been identified. On the other hand, Clusters 1 and 2 may have been considered as connected without the additional information gathered through genomic surveillance. Indeed, two cases from Cluster 2 (Z4187 and Z4145) were present in one of the bars where Cluster 1-related transmissions took place. Furthermore, the genomic data collected during the investigation of Cluster 2 could be analysed together with genomic data from the city of Barcelona to trace infection chains beyond Germany. The joint effort between researchers in Düsseldorf and Barcelona, which contributed to understanding the spread of the virus in Cluster 2, also demonstrates the potential of pan-European collaboration.

This study has multiple potential limitations. First, relevant cases may be missing from the analysis of the two clusters. Reasons for this may include undetected infections in asymptomatic individuals or failure to identify relevant cases during contact tracing or based on genetic data. Second, high-quality viral genomes were only available for ca 50% of SARS-CoV-2 cases in Düsseldorf during the considered period, so relevant cases in the genetic analysis may have been missed (see previous point). There remains also uncertainty with respect to cases for whom no sequencing data were available, such as the epidemiologically linked additional KP cases of Cluster 1. In addition, the assignment of KP1_5 to Cluster 1 remained ambiguous even with genetic data. Third, while the assignment of IP as the index case of Cluster 1 was supported by dates of symptom onset, the reconstructed pattern of putative transmission events in different bars, and the detection of related viral isolates from the Balearic Islands, a degree of uncertainty remained, as there was no way to rule out the presence of undetected cases or independent importation events at the beginning of the detected transmission chains. The detection of related sequences in other regions highlights the possibility of additional independent introduction events, in particular for Cluster 1 cases from mid-July onwards for which no link to IP could be identified, as well as the possibility of export of viral strains from the Düsseldorf area, as inferring the directionality of transmission from genetic data alone is generally not possible. In addition, it is possible that the structured case interviews failed to uncover relevant case travel histories. Fourth, the possibility of multiple exposures to SARS-CoV-2 during high-incidence periods, likely observed e.g. for two Cluster 2 cases in this study, represents a general challenge for the accurate tracing of transmission chains; inferred links that are not supported by both genomic and epidemiological evidence should be interpreted with caution. Fifth, the current study was carried out on a timescale of weeks and retrospective in nature; in the future, the sequencing speeds achievable with modern single-molecule sequencing technologies (from ‘swab to sequence’ in < 72 hours [10]) may enable implementations of IGS that support ‘real-time’ containment efforts. Sixth, the IGSD does not comprise the routine collection of clinical metadata, and case severity is not used as a sample selection criterion; inclusion of clinical metadata may further increase the utility of IGS.

Due to the emerging nature of IGS, there are many remaining open questions with respect to how to best design and implement an IGS system. For example, it is unclear at which level – national, regional, or at the level of a city – the integration between genetic and contact tracing data should be carried out, and how structured case interviews for backward contact tracing should best be conducted. The two clusters presented here clearly demonstrate the benefits of integrating local knowledge acquired ‘on-the-ground’ with data gathered by surveillance systems in different cities or countries; in the future, integration of local systems with larger national or European networks may enable the improved characterisation of introduction events and viral strain flow across cities and states.

Our work demonstrates the feasibility of tracing SARS-CoV-2 infection chains through a locally implemented system and during the later phases of the pandemic with high-incidence community transmission in an internationally connected city. This study complements existing studies from earlier phases of the pandemic [12,18] or from national or state-level genomic surveillance systems [7,11,13]. While the developed IGS system is currently limited to the tracing of SARS-CoV-2, its future potential applications include other emerging pathogens or multi-resistant bacterial pathogens.

## Supplementary Data

1170000 Supplementary Figure 1

## Supplementary Data

30900 Supplementary Note

## Supplementary Data

Supplementary Table 1

## Supplementary Data

4380000 30300 Supplementary Table 2

## Supplementary Data

Supplementary Table 3

## Supplementary Data

20400 20100 Supplementary Table 4

## Supplementary Data

21200 20100 Supplementary Table 5

## Supplementary Data

15000 Supplementary Table 6

## References

[1] World Health Organization (WHO). COVID-19 Weekly Epidemiological Update, Edition 106, published 24 August 2022. Geneva: WHO; 2022.

[2] Pung R Chiew CJ Young BE Chin S Chen MI Clapham HE Singapore 2019 Novel Coronavirus Outbreak Research Team . Investigation of three clusters of COVID-19 in Singapore: implications for surveillance and response measures. Lancet. 2020;395(10229):1039-46. 10.1016/S0140-6736(20)30528-6 32192580 PMC7269710

[3] Kang J Jang YY Kim J Han SH Lee KR Kim M South Korea’s responses to stop the COVID-19 pandemic. Am J Infect Control. 2020;48(9):1080-6. 10.1016/j.ajic.2020.06.003 32522606 PMC7834720

[4] Peak CM Kahn R Grad YH Childs LM Li R Lipsitch M Individual quarantine versus active monitoring of contacts for the mitigation of COVID-19: a modelling study. Lancet Infect Dis. 2020;20(9):1025-33. 10.1016/S1473-3099(20)30361-3 32445710 PMC7239635

[5] Kretzschmar ME Rozhnova G Bootsma MCJ van Boven M van de Wijgert JHHM Bonten MJM . Impact of delays on effectiveness of contact tracing strategies for COVID-19: a modelling study. Lancet Public Health. 2020;5(8):e452-9. 10.1016/S2468-2667(20)30157-2 32682487 PMC7365652

[6] Miller JS Bonacci RA Lash RR Moonan PK Houck P Van Meter JJ COVID-19 Case Investigation and Contact Tracing in Central Washington State, June-July 2020. J Community Health. 2021;46(5):918-21. 10.1007/s10900-021-00974-5 33689116 PMC7944242

[7] Lane CR Sherry NL Porter AF Duchene S Horan K Andersson P Genomics-informed responses in the elimination of COVID-19 in Victoria, Australia: an observational, genomic epidemiological study. Lancet Public Health. 2021;6(8):e547-56. 10.1016/S2468-2667(21)00133-X 34252365 PMC8270762

[8] COVID-19 Genomics UK (COG-UK) consortiumcontact@cogconsortium.uk . An integrated national scale SARS-CoV-2 genomic surveillance network. Lancet Microbe. 2020;1(3):e99-100. 10.1016/S2666-5247(20)30054-9 32835336 PMC7266609

[9] Callaway E . The coronavirus is mutating - does it matter? Nature. 2020;585(7824):174-7. 10.1038/d41586-020-02544-6 32901123

[10] Walker A Houwaart T Finzer P Ehlkes L Tyshaieva A Damagnez M German COVID-19 OMICS Initiative (DeCOI) . Characterization of Severe Acute Respiratory Syndrome Coronavirus 2 (SARS-CoV-2) Infection Clusters Based on Integrated Genomic Surveillance, Outbreak Analysis and Contact Tracing in an Urban Setting. Clin Infect Dis. 2022;74(6):1039-46. 34181711 10.1093/cid/ciab588 PMC8406867

[11] Hjorleifsson KE Rognvaldsson S Jonsson H Agustsdottir AB Andresdottir M Birgisdottir K Reconstruction of a large-scale outbreak of SARS-CoV-2 infection in Iceland informs vaccination strategies. Clin Microbiol Infect. 2022;28(6):852-8. 10.1016/j.cmi.2022.02.012 35182757 PMC8849849

[12] Popa A Genger JW Nicholson MD Penz T Schmid D Aberle SW Genomic epidemiology of superspreading events in Austria reveals mutational dynamics and transmission properties of SARS-CoV-2. Sci Transl Med. 2020;12(573):eabe2555. 10.1126/scitranslmed.abe2555 33229462 PMC7857414

[13] Douglas J Geoghegan JL Hadfield J Bouckaert R Storey M Ren X Real-Time Genomics for Tracking Severe Acute Respiratory Syndrome Coronavirus 2 Border Incursions after Virus Elimination, New Zealand. Emerg Infect Dis. 2021;27(9):2361-8. 10.3201/eid2709.211097 34424164 PMC8386796

[14] Katoh K Standley DM . MAFFT multiple sequence alignment software version 7: improvements in performance and usability. Mol Biol Evol. 2013;30(4):772-80. 10.1093/molbev/mst010 23329690 PMC3603318

[15] Shu Y McCauley J . GISAID: Global initiative on sharing all influenza data - from vision to reality. Euro Surveill. 2017;22(13):30494. 10.2807/1560-7917.ES.2017.22.13.30494 28382917 PMC5388101

[16] Rambaut A Holmes EC O’Toole Á Hill V McCrone JT Ruis C A dynamic nomenclature proposal for SARS-CoV-2 lineages to assist genomic epidemiology. Nat Microbiol. 2020;5(11):1403-7. 10.1038/s41564-020-0770-5 32669681 PMC7610519

[17] Robert Koch Institute (RKI). Informationen zur Ausweisung internationaler Risikogebiete, 23. Juli 2021. [Information on the Risk Classification of Regions outside Germany, 23 July 2021]. Berlin: RKI; 2021. Available from: https://www.rki.de/DE/Content/InfAZ/N/Neuartiges_Coronavirus/Transport/Archiv_Risikogebiete/Risikogebiete_2021-07-23.pdf?__blob=publicationFile

[18] Walker A Houwaart T Wienemann T Vasconcelos MK Strelow D Senff T Genetic structure of SARS-CoV-2 reflects clonal superspreading and multiple independent introduction events, North-Rhine Westphalia, Germany, February and March 2020. Euro Surveill. 2020;25(22). 10.2807/1560-7917.ES.2020.25.22.2000746 32524946 PMC7336109

[19] Letunic I Bork P . Interactive tree of life (iTOL) v3: an online tool for the display and annotation of phylogenetic and other trees. Nucleic Acids Res. 2016;44(W1):W242-5. 10.1093/nar/gkw290 27095192 PMC4987883

[20] Gansner ER North SC . An open graph visualization system and its applications to software engineering. Softw Pract Exper. 2000;30(11):1203-33. 10.1002/1097-024X(200009)30:11<1203::AID-SPE338>3.0.CO;2-N

[21] Campbell EM Boyles A Shankar A Kim J Knyazev S Cintron R MicrobeTrace: Retooling molecular epidemiology for rapid public health response. PLOS Comput Biol. 2021;17(9):e1009300. 10.1371/journal.pcbi.1009300 34492010 PMC8491948

