# Supplementary material for: Integrated genomic surveillance enables tracing of person-to-person SARS-CoV-2 transmission chains during community transmission and reveals extensive onward transmission of travel-imported infections, Germany, June to July 2021
Source: Euro Surveill. 2022 Oct 27;27(43):2101089. doi: 10.2807/1560-7917.ES.2022.27.43.2101089 (PMC9615415; doi:10.2807/1560-7917.ES.2022.27.43.2101089)
Supplement: Supplementary Figure 1 [file 2101089_SupplementaryFigure1.pdf]

This supplementary material is hosted by Eurosurveillance as supporting information alongside the article “Integrated genomic surveillance enables tracing of person-to-person SARS-CoV-2 transmission chains during community transmission and reveals extensive onward transmission of travel-imported infections, Germany, June to July 2021”, on behalf ofthe authors, who remain responsible for the accuracy and appropriateness of the content. The same standards for ethics, copyright, attributions and permissions as for the article apply. Supplements are not edited by Eurosurveillance and the journal is not responsible for the maintenance of any links or email addresses provided therein.

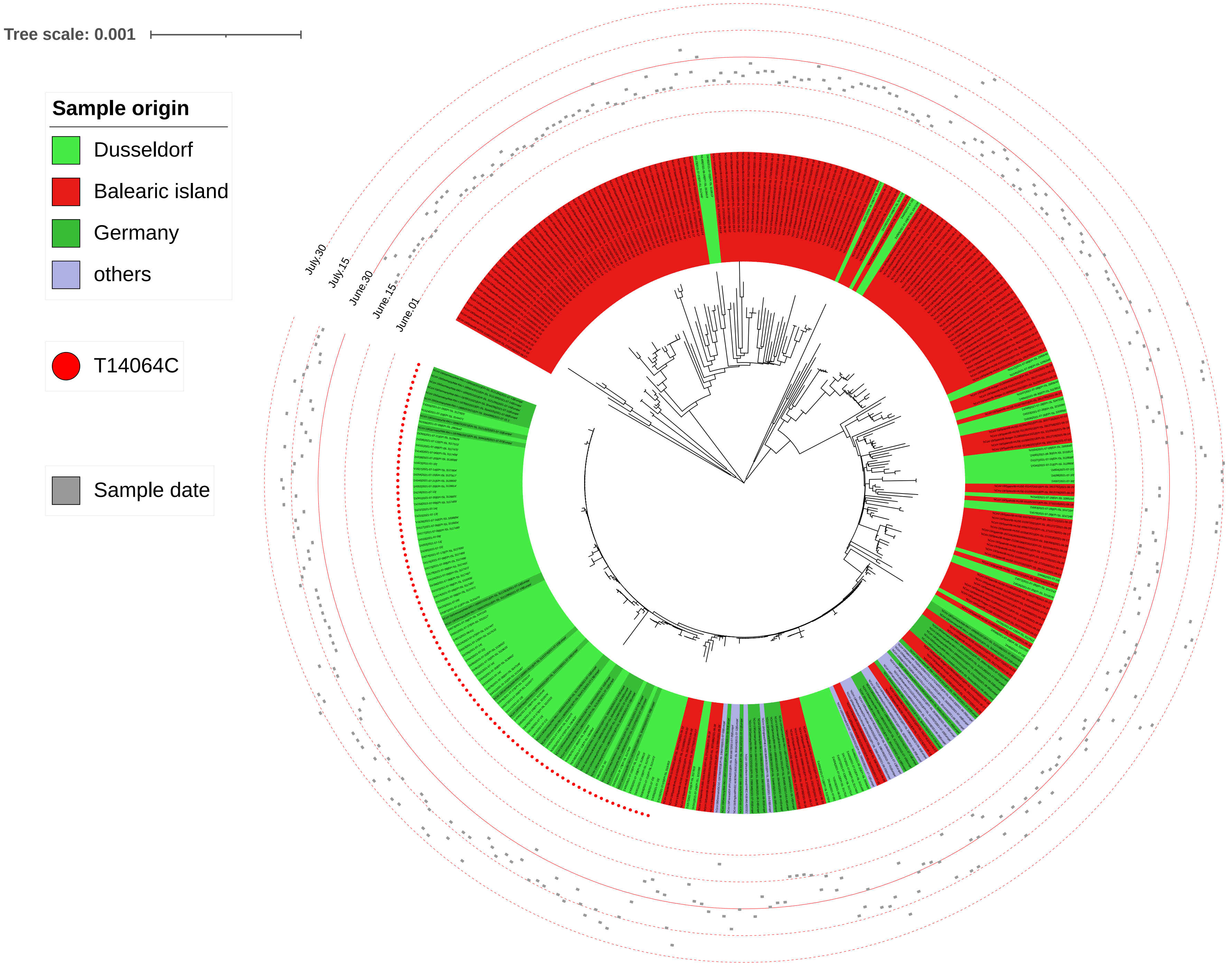

Supplementary Figure 1. Phylogenetic tree of IP and Cluster 1 sequences with contemporaneous isolate sequences from different localities. For phylogenetic analysis a phylogenetic tree was generated from the sequence of IP, 71 samples related to Cluster 1 from Düsseldorf (descendants of node I361 of the phylogenetic tree of the "extended" dataset used for the main phylogenetic analysis; see main text), 30 additional non-Cluster 1 random samples from Düsseldorf, 174 samples from Balearic islands collected between 14 June 2021 and 01 July 2021, and 61 related GISAID sequences sampled between 06 June and 15 July 2022 (see Methods section). The tree was generated with Geneious (see Methods) and visualized with iTol. Seven sequences were found to exhibit low-quality alignments in the multiple sequence alignment of the 347 input sequences by manual inspection and were removed from the alignment prior to construction of the tree. IDs are color-coded according to sample origin; Balearic Islands (red), Germany (green), Düsseldorf (light green) and other (blue). The presence of T14064C is indicated by red circles and the sampling time according to GISAID is shown in the radar blot. The sampling time given for IP (EPI ISL 3044996) is the sequencing date (2021-07-15) and not the date of positive PCR test (2021-07-05).
