# Supplementary material for: Integrated genomic surveillance enables tracing of person-to-person SARS-CoV-2 transmission chains during community transmission and reveals extensive onward transmission of travel-imported infections, Germany, June to July 2021
Source: Euro Surveill. 2022 Oct 27;27(43):2101089. doi: 10.2807/1560-7917.ES.2022.27.43.2101089 (PMC9615415; doi:10.2807/1560-7917.ES.2022.27.43.2101089)
Supplement: Supplementary Note [file 2101089_SupplementaryNote.pdf]

This supplementary material is hosted by *Eurosurveillance* as supporting information alongside the article “Integrated genomic surveillance enables tracing of person-to-person SARS-CoV-2 transmission chains during community transmission and reveals extensive onward transmission of travel-imported infections, Germany, June to July 2021”, on behalf of the authors, who remain responsible for the accuracy and appropriateness of the content. The same standards for ethics, copyright, attributions and permissions as for the article apply. Supplements are not edited by *Eurosurveillance* and the journal is not responsible for the maintenance of any links or email addresses provided therein.

Supplementary Note: Discussion on KP1_5 Cluster 1 membership.

With a genetic distance of 15 to the sequence of case IP of Cluster 1, the genome sequence of KP1_5 (N1515), despite clustering with Cluster 1 isolates, was removed as an outlier during the phylogenetic analysis. The sample was externally sequenced by one of the participating commercial diagnostic labs and the detected variants include an unusual pattern of two deletions of 6 nucleotides each, possibly indicating reduced quality of the assembled genomic sequence. The case was re-integrated into the analysis as a direct contact of KP_1 and KP_2. Despite its high genetic distance of 15, the viral sequence of KP1_5 carried the mutation T14064C, which was associated with Cluster 1 in the examined dataset, and which distinguished the sequence of IP from that of the identified genome from the Balearic Islands (GISAID accession EPI_ISL_2710175). In addition, increased genetic distances are sometimes observed in the analysis of epidemiologically clearly linked outbreak isolates [Walker et al. 2020, Walker et al. 2021]. The genetic data are therefore ambiguous with respect to the question sample KP1_5 belongs to the transmission chain originating with IP.

References

Walker, A., T. Houwaart, P. Finzer, L. Ehlkes, A. Tyshaieva, M. Damagnez, D. Strelow, A. Duplessis, J. Nicolai, T. Wienemann, T. Tamayo, M. Kohns Vasconcelos, L. Hulse, K. Hoffmann, N. Lubke, S. Hauka, M. Andree, M. P. Daumer, A. Thielen, S. Kolbe-Busch, K. Gobels, R. Zotz, K. Pfeffer, J. Timm, A. T. Dilthey and C.-O. I. German (2021). "Characterization of SARS-CoV-2 infection clusters based on integrated genomic surveillance, outbreak analysis and contact tracing in an urban setting." Clin Infect Dis.

Walker, A., T. Houwaart, T. Wienemann, M. K. Vasconcelos, D. Strelow, T. Senff, L. Hulse, O. Adams, M. Andree, S. Hauka, T. Feldt, B. E. Jensen, V. Keitel, D. Kindgen-Milles, J. Timm, K. Pfeffer and A. T. Dilthey (2020). "Genetic structure of SARS-CoV-2 reflects clonal superspreading and multiple independent introduction events, North-Rhine Westphalia, Germany, February and March 2020." Euro Surveill **25**(22).
